# Training strategies for a sustainable medical care: a survey among assistant and chief physicians in a tertiary care hospital in Germany

**DOI:** 10.1515/iss-2020-0024

**Published:** 2020-12-21

**Authors:** Juliane Kröplin, Eike-Ulrike Zauner, Hauke Dopp, Anke Forberger, Gerhard Schön, Reinhard Bschorer, Oliver Heese, Jörg-Peter Ritz

**Affiliations:** Department of Oral and Maxillofacial Surgery, Helios Kliniken Schwerin, Schwerin, Germany; Department of General and Visceral Surgery, Helios Kliniken Schwerin, Schwerin, Germany; Department of Cardiology, Helios Kliniken Schwerin, Schwerin, Germany; Depatment of Nephrology, Helios Kliniken Schwerin, Schwerin, Germany; Department of Medical Biometry and Epidemiology, University Medical Center Hamburg-Eppendorf, Hamburg, Germany; Department of Neurosurgery, Helios Kliniken Schwerin, Schwerin, Germany

**Keywords:** competency-based training, leadership, management, mentoring, surgical training, skills lab

## Abstract

**Purpose:**

As an essential part of the health care system, the requirements for specialist training are subject to a continuous process of change. The aim of the present study was to evaluate the current specialist training situation of all departments in a tertiary care hospital in Germany. Differences between assistant and chief physicians should be pointed out.

**Materials and methods:**

The analysis of the current training situation was carried out on the basis of an individually created questionnaire. The questionnaire content included career goal and specialism. The characteristic values initial training (IT), training structure (TS) and training content were measured on a numeric scale from 1 to 5. In addition, an overall assessment of the trainers’ competences was performed. The questionnaire was sent to 208 assistant physicians (AP) and 34 chief physicians (CP).

**Results:**

Totally 92 APs (44.2%) and 22 CPs (64.7%) participated. Senior physician was the most common career goal (34.1%), followed by the branch (28.6%). The importance of the topics initial training (IT) and training structure (TS) were evaluated as mean value: IT_CP_=1.5, IT_AP_=1.6; p=0.701 and TS_CP_=1.4, TS_AP_=1.5; p=0.669. The results concerning the implementation of the topics IT and TS in the daily routine show significant differences between APs and CPs (IT_CP_=2.0, IT_AP_=3.2; p=0.002; TS_CP_=1.9, TS_AP_=3.0; p<0.001). Skills lab training was acknowledged as the most important training format (CP=1.3, AP=1.5; p=0.401). The practical medical skills of the professional trainers were evaluated as high: AP: 94.6% (CP: 100.0%), as well as the training in interprofessional collaboration: AP: 79.4% (CP: 100.0%).

**Conclusion:**

Our data underline the importance of specialist training subjects. These are partly perceived very differently by APs and CPs. Innovative concepts for the induction phase, well-structured training curricula, providing management skills, the overall use of skills labs and digital documentation might support the satisfaction and the outcome of specialist training. This could also improve quality in patient care.

## Introduction

Specialist medical training is an essential part of the health care system in order to meet all the requirements of innovative medical care [1], [2]. Rapid technological and scientific medical progress requires high standards in the assessment and validation of competence acquisition in the professional education and training [1], [3], [4]. Additionally, the demographic change with an expected increase in multimorbid patients must be observed. The specialist training guidelines of Germany were amended by the German Medical Association in November 2018. The guidelines focus on a competency-based continuing professional development. The aim is to monitor the assistant physicians’ improvement in training continuously [5]. Demonstrating that the trainee has all competences to progress in postgraduate medical education, however, is challenging. It must be taken in to account that competence is no achievement. It is a lifelong learning process to gain knowledge, adapt to change and enhance overall performance [6], [7]. The carrier of surgeons depends to a large extend on these skills [8].

To date, there is no agreement on quality metrics and indicators for systematic medical education and training of healthcare professionals [9]. Within Europe postgraduate medical education is insufficient to promote high levels of professional competence throughout medical careers. As an essential component of specialist training, physicians should be supported in acquiring competences in continuous learning and assessment strategies. This includes the organization and management of activities and incentive structures for participation [10]. Innovative health care should be high in quality and cost effective while the health care system is becoming increasingly complex and costly [11]. This requires new competences and skills as well as innovative training strategies to meet the increasing future demands [1], [11].

The quality of training is the responsibility of the chief physicians and must be authorized regularly by the regional medical associations. In order to achieve both high quality in training and high quality in medical care, the satisfaction and motivation of assistant physicians plays a fundamental role [12].

The aim of the present study was to evaluate the current specialist training situation of all departments in a tertiary care hospital in Germany. The evaluation included the perspective of the assistant physicians, whereby in particular differences and similarities between assistant physicians and chief physicians were to be highlighted in the further education content.

## Materials and methods

### Study design and data collection

The present survey evaluates the importance and implementation of training subjects in a tertiary care hospital in Germany. The content includes career goal and the implementation and importance of the topics of initial training (IT), specialist training structure (TS) and training content. An overall assessment reflects the perception of the specialist trainers’ expertise concerning their medical and training competences. Inclusion criteria for participation was being assistant physician (AP) or chief physician (CP). The evaluation was conducted anonymously and voluntary. The current training situation was carried out on the basis of an individually prepared questionnaire. There was an adapted version for the chief physicians. The participation link was sent via the internal clinical e-mail account to all listed APs and CPs of the hospital. The data collection was carried out online based on the questionnaire.

### Questionnaire

The questionnaire was prepared by the representatives of the hospital’s assistant physicians. It was inspired by general issues of the local specialist training and based on satisfaction surveys concerning postgraduate medical education [13], [14]. First of all, the questionnaire contains items on the intended medical specialization and desired career goal. There were six medical specialist clusters: surgery (SURG; including all surgical specialties), internal medicine (INT), diagnostics (DIAG), anesthesiology/intensive care (ANA), neurology/psychiatry (PSY) und pediatrics (PED). The career goal was divided into clinical career (chief physicians, senior physicians, consultant) and non-clinical career (branch and miscellaneous).

On a numeric scale from 1 to 5, the importance (1=very important to 5=very not important) and implementation (1=completely implemented to 5=not implemented) of the characteristic values of initial training and training structure were assessed as arithmetic mean by the APs and CPs. Furthermore, the importance of five training contents was analyzed from 1 to 5 as well.

One part included the evaluation of the trainers’ medical and teaching competences rated as *complete* [4]*, rather yes* [3]*, rather not* [2] *and not* [1]*.*

The study has been reviewed by the Ethics committee of the medical faculty of University Rostock, Germany and has been approved (A 2018-0169).

### Statistics

All calculations were done using R Version 4.0.1. Statistical analysis was performed by unpaired, two-tailed Student’s t-test. A value of p<0.05 was considered statistically significant.

## Results

1. Demographics

**Figure 1:**
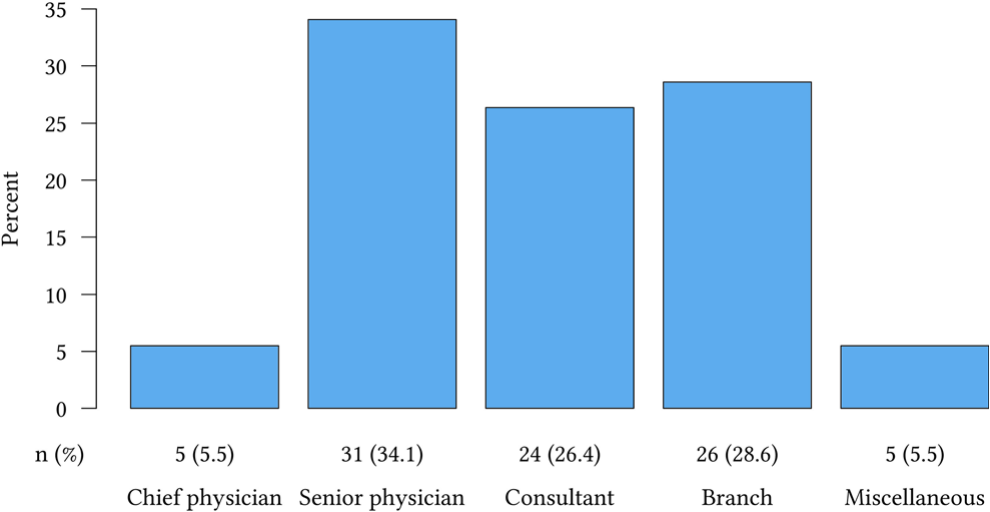
Carrere goals of the assistant physicians (n (%)).

The questionnaire was sent to 208 assistant physicians (AP) and 34 chief physicians (CP). The number of participants was 44.2% (n=92) for AP and 64.7% (n=22) for CP. 31.5% of the APs were in a specialist surgical training, 23.9% in specialist training for internal medicine, 12.0% for anesthesia/intensive care, 13.0% for diagnostics such as radiology, 3.3% for pediatrics and 16.3 for neurology/psychiatry. As presented in Figure 1, the most frequently long-term career goal was senior physician (34.1%), followed by branch (28.6%), consultant (26.4%), chief physician (5.5%) or another field of professional activity (5.5%). Four of the five APs who wanted to become chief physician belonged to the surgical group.

1. Importance and Implementation of initial training (IT) and training structure (TS)

**Figure 2:**
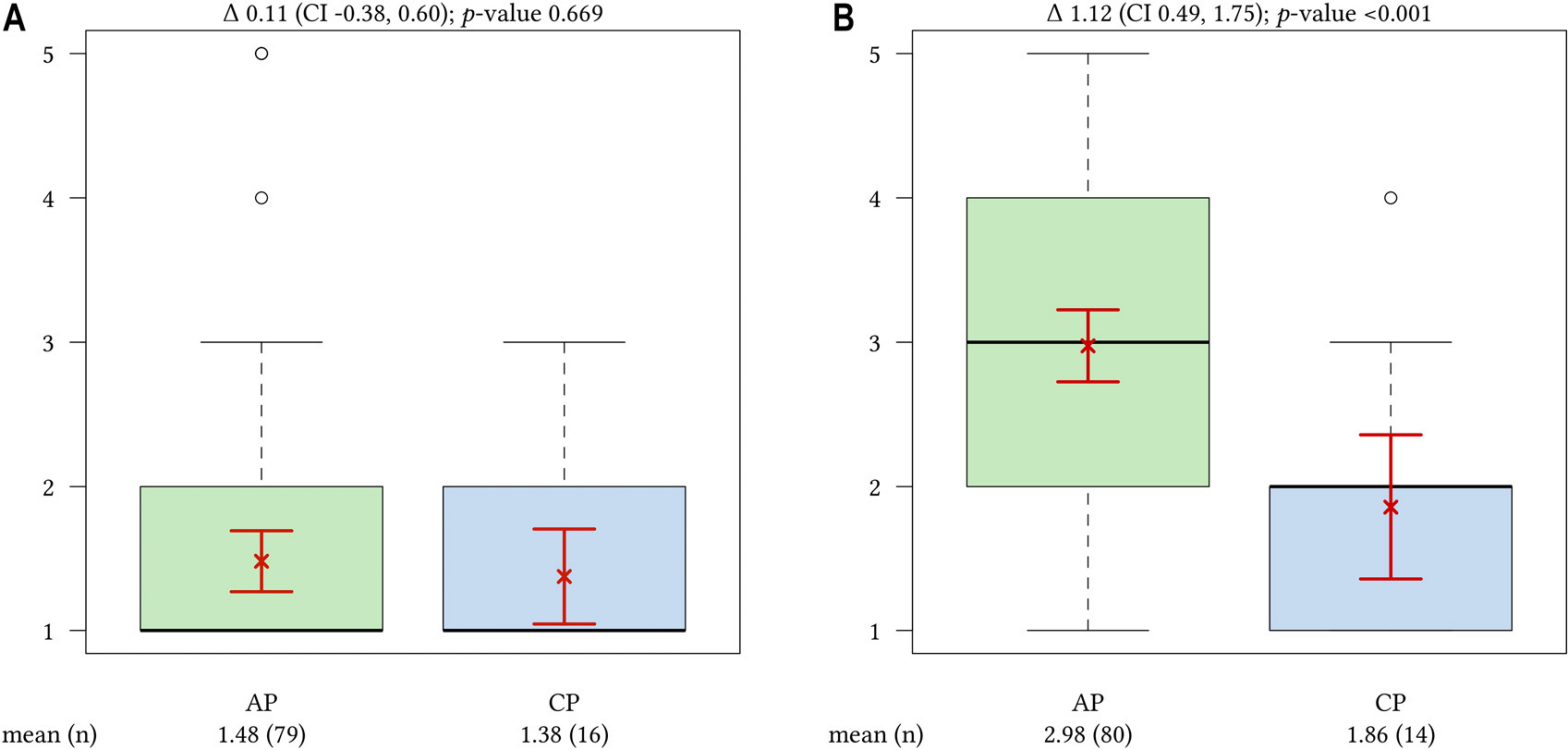
A/B Importance (A, left boxplot) and implementation (B, right boxplot) of the topic training structure, AP vs. CP. (mean (n). (1=very important/complete implemented – 5=very not important/not implemented) The arithmetic mean is represented as a cross and the confidence interval as horizontal lines.

Table 1. The importance of the topic of initial training (IT) was evaluated as high by APs and CPs (IT_CP_=1.5, IT_AP_=1.6; p=0.701). However, we observed significantly different values between APs and CPs regarding the implementation (IT_CP_=2.0, IT_AP_=3.2; p=0.002). Both groups assessed the training program structure as an important topic with no significant difference (TS_CP_=1.4, TS_AP_=1.5; p=0.669, Figure 2A). Again, there was also a significant difference between APs and CPs in the results regarding the implementation of the training structure (TS_CP_=1.9, TS_AP_=3.0; p≤0.001, Figure 2B).

1. Importance of training content

**Table 1:** Importance and Implementation concerning the topics initial training (IT) and training structure (TS) (%) AP vs. CP (1=very important/completely implemented – 5=very not important/ not implemented).

Importance and implementation of IT and TS, %	1	2	3	4	5	Mean	p-Values
AP IT	62.0	25.3	5.1	6.3	1.3	1.6	0.701
CP IT	56.2	37.5	6.2	0.0	0.0	1.5	
Importance							
AP TS	70.9	19.0	5.1	1.3	3.8	1.5	0.669
CP TS	68.8	25.0	6.2	0.0	0.0	1.4	
Importance							
AP IT	11.2	28.7	13.8	23.8	22.5	3.2	0.002
CP IT	21.4	57.1	21.4	0.0	0.0	2.0	
Implementation							
AP TS	10.0	22.5	38.8	17.5	11.2	3.0	<0.001
CP TS	35.7	50.0	7.1	7.1	0.0	1.9	
Implementation							

**Figure 3:**
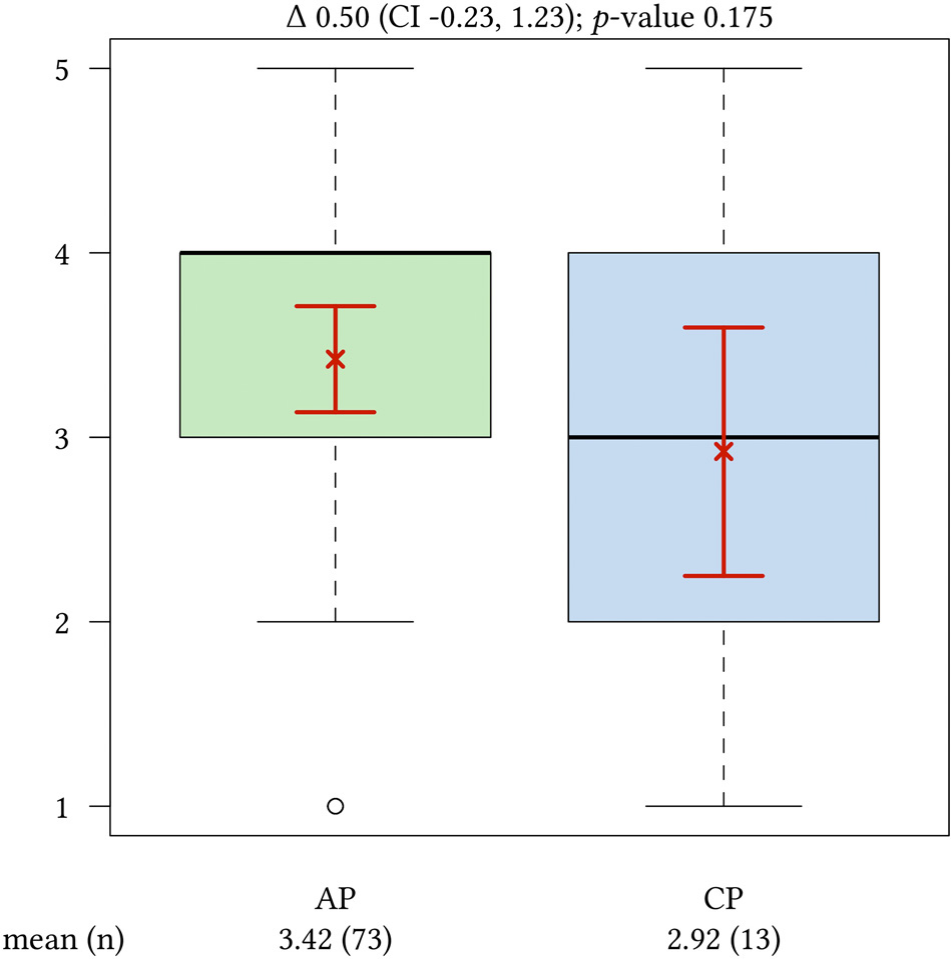
Importance of training content **Management**, AP vs. CP. (mean [n]). (1=very important – 5=very not important) The arithmetic mean is represented as a cross and the confidence interval as horizontal lines.

As presented in Table 2 the **skills lab** training was rated most important by APs and CPs (AP: 1.5, CP: 1.3; p=0.401). **Management** was valued as the least important training content: 3.4 (AP) and 2.9 (CP), p=0.175 (Figure 3). **Congress attendance** was assessed as 2.1 (AP) and 2.5 (CP); p=0.196). The values regarding the **morbidity and mortality conferences** show significant differences between APs (2.6) and CPs (1.7), p=0.012. As another important training subject **interdisciplinary courses** was valued as 2.2 (AP) and 1.9 (CP), p=0.237). Excluding the morbidity and mortality conference attendance all results show no significant differences between the both groups.

1. Trainers’ medical skills and teaching competences

**Table 2:** Importance of training content (%) AP vs. CP; 1=very important – 5=very not important.

Importance of training content, %	1	2	3	4	5	Mean	p-Values
AP	23.1	25.6	29.5	7.7	14.1	2.6	0.012
CP	50.0	28.6	21.4	0.0	0.0	1.7	
M & m conferences							
AP	33.8	37.5	18.8	5.0	5.0	2.1	0.195
CP	14.3	28.6	50.0	7.1	0.0	2.5	
Congress attendance							
AP	9.6	12.3	26.0	30.1	21.9	3.4	0.175
CP	7.7	30.8	30.8	23.1	7.7	2.9	
Management skills							
AP	26.0	41.6	23.4	3.9	5.2	2.2	0.237
CP	38.5	38.5	23.1	0.0	0.0	1.9	
Interdisciplinary courses							
AP	72.0	16.0	6.7	1.3	4.0	1.5	0.401
CP	75.0	25.0	0.0	0.0	0.0	1.3	
Skills lab training							

Table 3 shows the overall assessment regarding the trainers’ skills and their teaching competences. The skill best voted by both APs as well as CPs was the trainers’ **practical medical skills** (mean: 3.4 [AP] and 3.4 [CP] out of 4). Scientific work was rated as the worst teaching competence (mean: 2.2 [AP] and 2.8 [CP] out of 4). The values show significant differences between the trainers’ skills in **emergency management** (p=0.002) as well as teaching competence concerning **guideline adherence** (p=0.021), **scientific work** (p=0.013) and **interprofessional collaboration** (p=0.012).

**Table 3:** Trainers’ competences (%) AP vs. CP (4=complete – 1=not).

Trainers competences, %	Complete	Rather yes	Rather not	Not	Mean	p-Values
	(4)	(3)	(2)	(1)		
AP	13.0	48.9	27.2	10.9	2.6	0.002
CP	45.5	45.5	9.1	0.0	3.4	
Emergency management						
AP	24.2	48.4	25.3	2.2	3.0	0.021
CP	54.5	40.9	4.5	0.0	3.5	
Teaching guideline adherence						
AP	26.1	53.3	18.5	2.2	3.0	0.012
CP	59.1	40.9	0.0	0.0	3.6	
Supporting interprofessional collaboration						
AP	7.7	26.4	40.7	25.3	2.2	0.013
CP	22.7	40.9	36.4	0.0	2.9	
Teaching scientific work						
AP	31.9	50.5	17.6	0.0	3.1	0.104
CP	33.3	66.7	0.0	0.0	3.3	
Knowledge transfer						
AP	19.6	42.4	34.8	3.3	2.8	0.064
CP	38.1	52.4	9.5	0.0	3.3	
Teaching teamwork						
AP	45.1	50.5	4.4	0.0	3.4	0.581
CP	42.9	57.1	0.0	0.0	3.4	
Practical medical skills						
AP	23.9	39.1	30.4	6.5	2.8	0.152
CP	42.9	42.9	14.3	0.0	3.3	
Giving timely feedback						

## Discussion

There is a growing demand for innovative strategies that support continuing professional development, structure and adherence in specialist training. Especially the features of surgical training present a challenge [8]. The need for these adjustments is due to demographic changes and economic issues, as well as the rapid technical and scientific progress in medical care [1], [3], [15]. Additionally, the current specialist training situation in Germany is partly perceived as unstructured, unpredictable and associated with dissatisfaction by the junior physicians [14], [16]. Satisfaction surveys have shown that especially the young surgeons call for objective, transparent and fair operation planning which is coherent with the operation catalogues of the regional medical associations. These issues are of high importance considering that a great satisfaction with the training program also could provide a high quality in training and patient care [17], [18]. Furthermore, demographic changes are leading to increasing competition for the high potentials of the younger medical generation. Especially the surgical disciplines are requested to create training concepts and working conditions in order to keep up in the “war for talents” and to maintain a high quality of medical care [17], [19].

The aim of the present study was to evaluate the perception of the current training situation of all departments in a tertiary care hospital in Germany from the assistant physicians’ point of view. Additionally, all chief physicians of the hospital were asked attend the survey in an adapted version. The evaluation was supposed to analyze both perspectives. The intention was to support better communication between APs and CPs in order to provide sustainable training conditions.

### Career vs. work-life balance?

In 2019 the German Medical Association published its results on “doctors’ statistics” [20]. Based on these data, there is a predominant number of physicians working in hospitals in Germany (51.5%). This supports our results, in which the majority of participants also indicated their intention to pursue a medical career at a hospital as well (66.0%). The most desirable career goal in our survey is the senior physician. A total of 39.6% of the assistant physicians desires a leading position. But actually only 16.1% of doctors in Germany are working as leading physicians at a hospital. Our results support the willingness of the attending APs to assume responsibility as a leading physician – but not at the forefront. Only 5.5% of them want to become chief physician.

Our data support that there are remarkable similarities in the values concerning the importance of initial training and training structure as well as the importance of established training events and themes. This confirms that both APs and CPs are aware of the tremendous meaning of actively contribution to specialist training. But there are also striking differences between both perspectives. This might be attributable to different expectations and perception of the framework that constitute an excellent specialist training and patient care. A great difference in experience between APs and CPs should be considered as well. Today, the junior physicians call for working conditions that respect issues of “work-life balance” such as regular working hours, part time opportunities and less overtime hours [21]. These demands in combination with Working Hours Act restrictions typically lead to conflicts with the CPs who have to meet all training requirements defined by the extensive training curricula of the regional medical associations. Our findings support the view that the induction phase and a well-structured training program is of great importance for APs and CPs. It can be assumed that a well-structured training program developed at the beginning of the training might clarify similarities and differences in the expectations. Individual training and career goals should be considered as well. Especially within the induction phase APs as well as CPs might profit from Mentoring programs. Mentoring encourages the professional development of individuals [22]. Additionally, there is increasing evidence for better training outcomes supported by mentoring [23]. Digital documentation of the training content and progress might also improve training outcome. Haag et al. [24] announced the use of existing or still to be collected data to better adapt teaching to the actual needs as used in Learning Analytics and Educational Data Mining. To generate the highest value of digitization, physicians should play a more active and positive role [15].

### Best practice: which training content do we need?

Regarding the required specialist training content, there are remarkable similarities between APs and CPs. Morbidity and mortality (m & m) conferences, congress attendance, skills lab training and interdisciplinary courses were predominantly valued important by APs and CPs. Except participation in m & m conferences, the values show no significant difference between both groups. The application of skills lab training is valued as most important training content by APs and CPs.

According to the current literature, there is an increasing importance and growing use of skills lab training to provide clinical skills. Severe surgical interventions, emergency management and complex intensive care procedures can be trained [25], [26], [27], [28]. A comprehensive available training program based on virtual reality simulation-based training could provide skills in teamwork, improve knowledge, technical skills and managing complex procedures [25], [29], [30]. Especially virtual reality simulations may improve surgical education and training by offering novel opportunities, for instance regarding the assessment of surgical competences by detailed feedback and measurements of the trainee’s performance [31]. However, economic issues must be considered. In order to provide easy access to simulation training, there is a further need to share resources and reduce costs [27]. In 2008 Sturm et al. [32] showed in a systematic review that skills acquired by simulation-based training can be successfully transferred to the operative setting. It seems evident to conclude that simulation training offers new opportunities to maintain a high quality in training notwithstanding an increasing workload, especially in the operation theatres. New technologies with further developments in simulation-based virtual reality training might support the well-known benefits of simulation training.

The least important training content for APs and CPs is management. Not even a third of all APs confirm that management is an important training content. This is remarkable with respect to our results that 39.6% of APs are planning a career as a medical leader at hospital. Additionally, it can be assumed that also a part of the branch and miscellaneous group will work in a leading position and will assume management tasks after finish specialist training in hospital. Training skills concerning management – including leadership competences – were underrepresented in the medical education and specialist training for a long time. But things are certainly moving. The National Learning Objectives Catalogue in Medicine (NKLM) was adopted in June 2015. In order to link medical education with postgraduate training, competences in management, communication, interdisciplinary and interprofessional collaboration and scientific work were implemented [4], [33]. Actually, there is little evidence of better performance of medical managers compared to non-medical managers [34]. Xirasagar et al. [35], [36] described a more effective competence in leadership of physicians who had graduated in management training such as Masters of Business Administration (MBA). In 2018 the Harvard Business Review published an article entitled “Why doctors need leadership training” [37]. Here, Rotenstein et al. describe the growing evidence of the positive influence of leadership skills and management practices on patient and healthcare organization outcomes. This includes lower mortality and better financial performance. This competence is already demanded in the first years of graduate medical training by the necessity to lead teams of less experienced physicians and other care personnel. Leadership skills are described as knowing how to lead a team, how to confront problematic employees, how to coach and develop others and how to resolve conflicts. Rotenstein et al. also announced the increasing costs of training and assessment enhancing leadership skills. It seems evident to conclude, that training strategies including the improvement of management practice and leadership skills might have a positive influence on specialist training.

### Quality in medical care: a top priority

Our results indicate that both groups, APs and CPs, generally perceive a high level of competence of the specialist trainers. But there are some significant differences between APs and CPs. This regard managing emergencies, teaching competences in guideline-based patient care, scientific work and supporting interprofessional collaboration.

It must be considered that the various specialist groups have different priorities in their training programs. This observation is supported by the different contents of the specialist training guidelines. For example, training for the future anesthesiologists is much more focused on emergencies compared to the diagnostic group. Nevertheless, the management of emergencies is a crucial competence for all physicians, independent from the status. It should be carried out regularly by trainers of all departments.

The least valued trainer competence is the teaching of scientific work, confirmed by the CPs, but with significant difference. It is reasonable to assume that this is due to the non-university status of the clinic at the time of the survey.

The general content of the specialist training guidelines of Germany require the competence of guideline application [38]. Our findings show that the majority confirmed the trainers’ competence in teaching guideline adherent knowledge and skills. However, there is also a significant difference between the groups. Guideline adherence is often used as an indicator of quality in medical care [33]. In a comprehensive study in the US McGlynn et al. [39] showed that only about a half of all patients (55.0%) received the medical care recommended in guidelines.

It can be assumed that the actual training content is predominantly focused on traditional medical competences. In line with this observation, our questionnaire results in both groups confirm a high level of satisfaction with the practical medical skills of the trainers and the transfer of medical knowledge. Reznick et al. [25] described appropriate workload, good supervision of the practice and good quality feedback as key factors. More than a third of the assistant physicians interviewed in our survey are (rather) dissatisfied with the provision of timely feedback. In a quality approach to examining differences in high and low performance facilities, Hysong et al. [40] found the delivery of feedback in high performance facilities to be timely, individualized and non-punitive. Timely feedback dialogues in a targeted context following new supervised training sessions might support satisfaction and progress in training by improving the performance of assistant physicians. Both established and innovative teaching concepts based on instruction (e.g. Peyton’s Four-Step Approach), deliberate practice and timely feedback might provide these demands [28], [31]. As described in the literature, training methods such as OSATS (Objective Structured Assessments of Technical Skills) and VR (Virtual Reality) systems have a positive influence on the objective assessment of technical skills in surgery [31].

However, it can be assumed that the training of management competences is highly connected to the competence of training skills supporting teamwork and interprofessional collaboration as well as a good quality of feedback dialogues. A stronger focus on management and leadership competences might also provide higher satisfaction with these training skills. Additionally, good communication between both groups could enable a more constructive exchange, show differences and solutions and thus could be an important component for successful specialist training.

An induction phase supported by mentoring programs, well-structured training curricula, the overall use of skills lab training, timely feedback dialogues and the digitization of training content and progress might support satisfaction among assistant physicians and a high quality of specialist training. Additionally, an internal comparison of the AP clusters within the framework of a multicentre study design might support a discipline-specific structure.

More quality analysis and research are needed to identify the determination of assistant physician satisfaction and to develop strategies to improve postgraduate training quality.

## Conclusion

Specialist medical training is an essential part of the health care system to manage innovative medical care. Our results demonstrate the importance of issues concerning specialist training in a tertiary care hospital in Germany. The effectiveness and implementation of the training as well as the structuring of the training program is partly perceived very differently by chief physicians and assistant physicians. The subjective perception of an unstructured training program and insufficient initial training by assistant physicians is not confirmed by the chief physicians. An implementation of procedures and concepts that ensure both training effectiveness and structuring might also improve satisfaction in both groups. From the authors’ point of view, this could be realized through:

– Projects for better communication between CPs and APs like biannual training dialogues and flexible, timely feedback dialogues after new training sessions.
– Adherence for the induction phase (supported by mentoring-programs).
– Well-structured training curricula with flexible individualized opportunities.
– Improvement of management competences for trainers and trainees.
– Enhancing access to simulation training with supervision settings.
– Regular reviews of practical and theoretical competences.
– Digital documentation of the training content and progress.
