# Supplementary material for: Training strategies for a sustainable medical care: a survey among assistant and chief physicians in a tertiary care hospital in Germany
Source: Innov Surg Sci. 2020 Dec 21;5(3-4):81–9. doi: 10.1515/iss-2020-0024 (PMC7790178; doi:10.1515/iss-2020-0024)
Supplement: Supplementary file 1 [file iss-05-20200024-s001.pdf]

Reviewer Assessment

Juliane Kröplin et. al: Training strategies for a sustainable medical care. A survey among assistant and chief physicians in a tertiary care hospital in Germany

Reviewers' Comments to Original Submission

Reviewer 1: Schröder, Wolfgang

Date received: 01-Oct-2020
Reviewer recommendation: **Return to author for minor modifications**
Reviewer overall scoring: **High**

Assessment Form scores: 5 = High/Yes; 3 = Medium/Adequate; 1 = Low

Is the subject area appropriate for the journal		4			
Does the title clearly reflect the paper's content?		4			
Does the abstract clearly reflect the paper's content		4			
Do the keywords clearly reflect the paper's content?				2	
Does the introduction present the problem clearly?	5				
Are the results/ conclusions justified?		4			
How comprehensive and up-to-date is the subject matter presented?		4			
How adequate is the data presentation?		4			
Are units and terminology used correctly?		4			
Is the number of cases adequate?				2	
Are the experimental methods/ clinical studies adequate?		4			
Is the length appropriate in relation to the content?		4			
Does the reader get new insights from the article?					
Please rate the practical significance.			3		
Please rate the accuracy of methods.		4			
Please rate the statistical evaluation and quality control.		4			
Please rate the appropriateness of the figures and tables.		4			
Please rate the appropriateness of the references.	5				
Please evaluate the writing style and use of language.		4			
Please judge the overall scientific quality of the manuscript.		4			
Are the methods used worthy of reproduction in greater deal?	Yes				
Would you be willing to review a revision of this manuscript?	No				

Comments to author: In general, the manuscript is very well written and can be publish with minor revisions. The data of this questionnaire support several other surveys and point out to the necessity of structure specialist training for all medical disciplines.
Page 4, line 37: 6 and not 5 medical specialist clusters
In this context, the reader would be interested if there are differences between the two main groups of surgical and internal medical AP's and whether the training for these groups should be structured and organized differently.

Reviewer 2: anonymous

Date received: 24-Oct-2020
Reviewer recommendation: **Return to author for minor modifications**
Reviewer overall scoring: **High**

Assessment Form scores: 5 = High/Yes; 3 = Medium/Adequate; 1 = Low

Is the subject area appropriate for the journal	5				
Does the title clearly reflect the paper's content?	5				
Does the abstract clearly reflect the paper's content		4			
Do the keywords clearly reflect the paper's content?	5				
Does the introduction present the problem clearly?	5				
Are the results/ conclusions justified?	5				
How comprehensive and up-to-date is the subject matter presented?		4			
How adequate is the data presentation?	5				
Are units and terminology used correctly?		4			
Is the number of cases adequate?		4			
Are the experimental methods/ clinical studies adequate?	5				
Is the length appropriate in relation to the content?	5				
Does the reader get new insights from the article?		4			
Please rate the practical significance.		4			
Please rate the accuracy of methods.	5				
Please rate the statistical evaluation and quality control.	5				
Please rate the appropriateness of the figures and tables.	5				
Please rate the appropriateness of the references.	5				
Please evaluate the writing style and use of language.			3		
Please judge the overall scientific quality of the manuscript.		4			
Are the methods used worthy of reproduction in greater deal?	Yes				
Would you be willing to review a revision of this manuscript?	Yes				

Comments to author: Congratulation, good work. Please just control the English grammar, vocabulary and syntax, e.g. by a native speaker.

Authors' Response to Reviewer Comments

Date received: 01-Nov-2020

Response to reviewer 1

In general, the manuscript is very well written and can be publish with minor revisions. The data of this questionaire support several other surveys and point out to the neccesity of structure specialist training for all medical diciplines.

1. Page 4, line 37: 6 and not 5 medical specialist clusters

Thank you very much for your review. We have corrected the number of clusters accordingly.

2. In this context, the reader would be interested if there are differencies between the two main groups of surgical and internal medical AP's and whether the training for these groups should be structured and organized differently.

This is a very exciting topic, which we have already discussed before submission. Due to the unicentre study design we decided against an internal comparison in the AP group. In this way, we want to preserve the anonymity of the participants and concentrate on the study objective of a comparison between chief physicians and assistant physicians.

We have added the following sentence:

"An internal comparison of the AP clusters within the framework of a multicentre study design might support a discipline-specific structure."

Response to reviewer 2

Congratulation, good work. Please just controll the english grammar, vocabulary and syntax, e.g. by a native speaker.

Thank you very much for your review. We have controlled and adjusted the English grammar, vocabulary and syntax.

Reviewers' Comments to Revised Submission

Reviewer 2: anonymous

Date received: 07-Nov-2020
Reviewer recommendation: **Accept in present form**
Reviewer overall scoring: **High**

Assessment Form scores: 5 = High/Yes; 3 = Medium/Adequate; 1 = Low

Is the subject area appropriate for the journal		4			
Does the title clearly reflect the paper's content?	5				
Does the abstract clearly reflect the paper's content		4			
Do the keywords clearly reflect the paper's content?		4			
Does the introduction present the problem clearly?		4			
Are the results/ conclusions justified?		4			

How comprehensive and up-to-date is the subject matter presented?		4			
How adequate is the data presentation?	5				
Are units and terminology used correctly?		4			
Is the number of cases adequate?	5				
Are the experimental methods/ clinical studies adequate?		4			
Is the length appropriate in relation to the content?		4			
Does the reader get new insights from the article?			3		
Please rate the practical significance.		4			
Please rate the accuracy of methods.		4			
Please rate the statistical evaluation and quality control.		4			
Please rate the appropriateness of the figures and tables.		4			
Please rate the appropriateness of the references.		4			
Please evaluate the writing style and use of language.		4			
Please judge the overall scientific quality of the manuscript.		4			
Are the methods used worthy of reproduction in greater deal?					
Would you be willing to review a revision of this manuscript?	Yes				

Comments to author: Good work.

Comments by the Editor-in-Chief to Revised Submission

All reviewer comments have been addressed in the revised version of this manuscript. Therefore we refrain from inviting a new reviewer. Reviewer 1 was not available for evaluation of the revised version.
